# Regulation of Hippo signaling and triple negative breast cancer progression by an ubiquitin ligase RNF187

**DOI:** 10.1038/s41389-020-0220-5

**Published:** 2020-03-20

**Authors:** Zhonghao Wang, Qiong Kong, Peng Su, Miao Duan, Min Xue, Xin Li, Jianing Tang, Zhitao Gao, Beibei Wang, Zhongbo Li, Yun Liu, Xiao Yang, Ruilin Cao, Tingting Song, Ke Wang, Yuqing Cai, Danfeng Wu, Jinglei Li, Gaosong Wu, Asha M. Guled, Jian Zhu, Cheng Yan, Ting Zhuang

**Affiliations:** 1 Henan Key Laboratory of Immunology and Targeted Therapy, School of Laboratory Medicine, Henan Collaborative Innovation Center of Molecular Diagnosis and Laboratory Medicine, Xinxiang Medical University, 453003 Xinxiang, Henan Province P.R. China; 2 School of Stomatology, Xinxiang Medical University, 453003 Xinxiang, Henan Province P.R. China; 3 School of International Education, Xinxiang Medical University, 453003 Xinxiang, Henan Province P.R. China; 4 Department of Pathology, Qilu Hospital, Shandong University, Jinan, P.R. China; 5 School of Basic Medical Science, Shandong University, Jinan, P.R. China; 6 Department of Thyroid and Breast Surgery, Zhongnan Hospital of Wuhan University, Wuhan, China; 7 Department of Radiology, Guangdong Provincial People’s Hospital, Guangdong Academy of Medical Science, Guangzhou, P.R. China; 8 School of Medicine, Wuhan University, Wuhan, China; 9 School of Medicine, Xinxiang University, 453003 Xinxiang, Henan P.R. China

**Keywords:** Breast cancer, Cell signalling

## Abstract

Breast cancer is the most common malignancy for women worldwide, while Triple Negative Breast Cancer (TNBC) accounts for 20% in all patients. Compared with estrogen receptor positive breast cancer, which could be effectively controlled via endocrine therapy, TNBC is more aggressive and worse in prognosis. It is therefore urgent and necessary to develop a novel therapeutic strategy for TNBC treatment. Recent studies identified Hippo signaling is highly activated in TNBC, which could be a driving pathway for TNBC progression. In our study, we determine RNF187 as a negative regulator for Hippo signaling activation. RNF187 depletion significantly decreases cell migration and invasion capacity in TNBC. These effects could be rescued by further YAP depletion. Depletion of RNF187 increases the YAP protein level and Hippo signaling target genes, such as CTGF and CYR61 in TNBC. Immuno-precipitation assay shows that RNF187 associates with YAP, promoting its degradation possibly via inducing YAP K48-dependent poly-ubiquitination. Interestingly, Our clinical data reveals that RNF187 reversely correlates with YAP protein level and Hippo target genes. RNF187 tends to correlate with good prognosis in TNBC patients. Our study provides evidence to establish a proteolytic mechanism in regulation Hippo signaling activation in TNBC.

## Introduction

Triple negative breast cancer^1^ (TNBC) is the most aggressive^2,3^ subtype of breast cancer, which harbors high genetic heterogeneity^4^. Compared with hormone receptor positive breast cancer, TNBC has a high rate of genomic mutation, gene amplification, and deletion^5^. It harbors different kinds of tumor suppressive gene mutations including P53 and Rb. Thus, it is still lack of effective “targeted” therapies in triple negative breast cancer, making it urgent to understand the biological effect and novel therapeutic targets for TNBC^6^.

The control of tissue growth and organ size depends on a balance between cell proliferation and cell death, which is tightly controlled by both systematic and organ intrinsic mechanisms^7^. Hippo pathway is firstly identified from genetic screening in Drosophila, which is a novel and evolutionary conserved tumor suppressor pathway. Hippo pathway controls the tissue growth and organ size by simultaneously restricting cell growth and cell proliferation while promoting cell death. The core Hippo pathway consists of a kinase cascade: the upstream kinase MST1/2 activates a downstream kinase LATS1/2, leading to phosphorylation and inactivation of a transcriptional cofactors YAP/TAZ^8^. YAP protein is the most important downstream activator for Hippo signaling, which shuttle between the cytoplasm and nucleus. When Hippo pathway is activated, YAP associates with transcriptional factors and functions as a transcriptional co-activator to promote Hippo target gene expression, including CTGF and CYR61^9^.

The abnormality of Hippo signaling was reported in several human cancers. For example, YAP gene amplification was found in liver cancer and TNBC^10,11^. Besides, experimental studies revealed that YAP controlled several cancers’ biological behaviors, such as carcinogenesis, cell survival and “stemness” maintenance^12,13^. In TNBC, population based studies showed that Hippo signaling activation related to TNBC breast cancer risk^14^. YAP gene amplification specifically existed in TNBC, not in estrogen receptor alpha positive breast cancer (https://www.cbioportal.org). Besides, depletion of YAP in TNBC cell lines inhibited cell invasion and proliferation in both in vitro and in vivo^3,15–17^. All these studies indicated that Hippo activation is an important driving force for TNBC progression. Since YAP signaling was shown to be critical in cancer progression, targeting YAP could be an appealing strategy for TNBC. A few studies suggested verteporfin could block YAP-TEAD interaction in experimental cancer models^18^. However, due to many obstacles in target YAP-TEAD interactions, directly abrogate the YAP-TEAD association is still premature in clinical application.

RNF187 (Ring finger 187), also named as RING domain AP-1 co-activator-1 (RACO-1), is one of the RING family members^19^. RNF187 functions as an E3 ubiquitin ligase in modulation cellular biological processes. Previous studies showed that RNF187 is required for AP-1 mediated cell proliferation^20^. Besides recent studies showed that RNF187 could play an oncogenic role in several cancers^20–23^. For example, RNF187 could promote liver cancer progression via Norch1 signaling^21^. However, in our current study, RNF187 functions as a tumor suppressor in TNBC progression. RNF187 promotes YAP protein K48 linked poly-ubiquitination, which subsequently leads to YAP degradation and transcriptional repression of Hippo target genes in TNBC.

## Results

### RNF187 inhibits migration and invasion in triple negative breast cancer cells

**Fig. 1:**
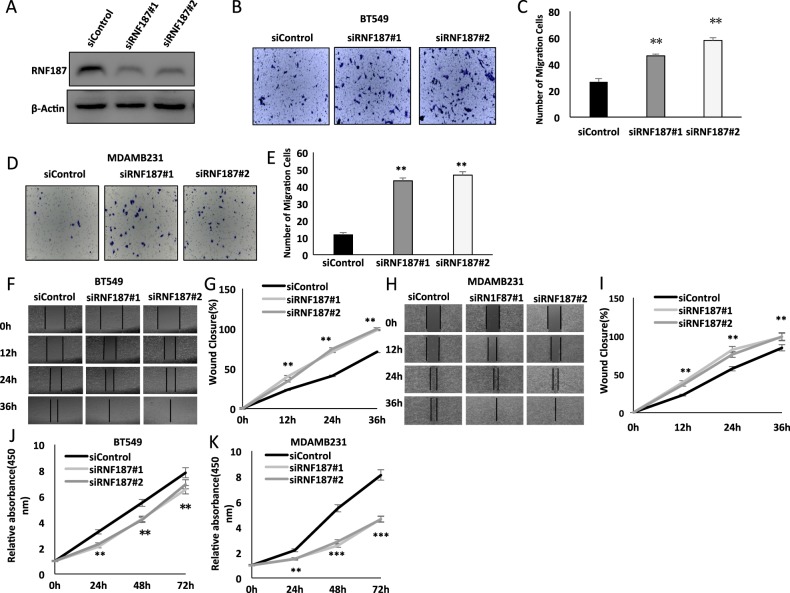
RNF187 inhibits migration and invasion in triple negative breast cancer cells. **a** RNF187 knocking down efficiency in TNBC cell lines. BT549 cells were transfected with RNF187 siRNA. The knockdown efficiency was measured via western blot. **b**, **c** RNF187 depletion promotes TNBC cell migration in BT549 cells. Two independent siRNA were used in the study. Transwell was used to check the migration capacity. The cell number was counted and Data are presented as ±SD. **P < 0.01, ***P < 0.001 (student’s *t*-test). **d**, **e** RNF187 depletion promotes TNBC cell migration in MDAMB231 cells. Two independent siRNA were used in the study. Transwell was used to check the migration capacity. The cell number was counted and Data are presented as ±SD. **P < 0.01, ***P < 0.001 (student’s *t*-test). **f**, **g** Wound-healing assay of BT549 cells were transfected with indicated 50 nM RNF187 siRNA (mix of #1 and #2) or 50 nM control siRNA. Quantification of wound closure at the indicated time points. Data are presented as ± SD. **P < 0.01, ***P < 0.001 (student’s *t*-test). **h**, **i** Wound-healing assay of MDAMB231 cells were transfected with indicated 50 nM RNF187 siRNA (mix of #1 and #2) or 50 nM control siRNA. Quantification of wound closure at the indicated time points. Data are presented as ± SD. **P < 0.01, ***P < 0.001 (student’s *t*-test). **j**, **k** RNF187 depletion inhibits TNBC cancer cell proliferation. BT549 and MDAMB231 cells were transfected with siControl or siRNF187. After 24 h, the WST assay was used to determine the cellar metabolic activity at indicated time points after infection. Experiments were done in triplicates. *P < 0.05; **P < 0.01; ***P < 0.001 for cell growth comparison.

In order to investigate the role of RNF187 in triple negative breast cancer cells, we utilized BT549 and MDAMB231 cells to carry out most of the experiments. To avoid off-target effects, we used two different siRNA with high knocking-down efficiency (Fig. 1a). The trans-well assay shows that RNF187 depletion increases the number of invasive cells both in BT549 and MDAMB231 cells (Fig. 1b–e). Besides, the wound healing experiments demonstrate that siRNF187 promotes cancer cell migration capacity in both BT549 and MDAMB231 cells (Fig. 1f–i). However, RNF187 depletion shows inhibition of cell proliferation in these two cancer cells by WST-1 experiments, which is consistent with previous studies^19^ (Fig. 1j, k).

### RNF187 inhibits Hippo signaling in triple negative breast cancer cells

**Fig. 2:**
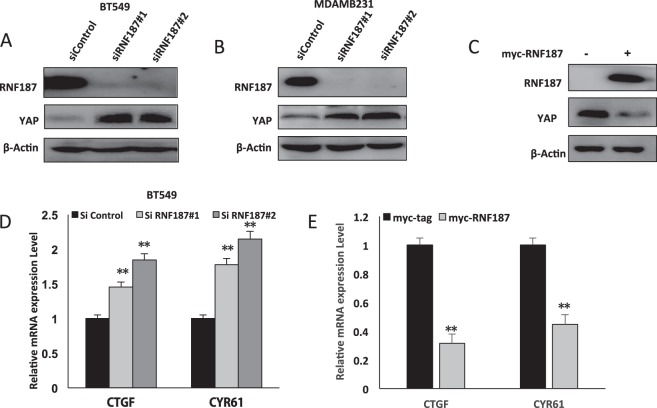
RNF187 inhibits Hippo signaling in triple negative breast cancer cells. **a** RNF187 depletion increased YAP protein levels in BT549 cells. BT549 cells were transfected with siControl or siRNF187. After 48 h, cells were harvested for western blot analysis. RNF187 and YAP protein levels were determined by Western blot. Actin was used as internal control. **b** RNF187 depletion increased YAP protein levels in MDAMB231 cells. MDAMB231 cells were transfected with siControl or siRNF187. After 48 h, cells were harvested for western blot analysis. RNF187 and YAP protein levels were determined by Western blot. Actin was used as internal control. **c** RNF187 over-expression decreased YAP protein levels in BT549 cells. BT549 cells were transfected with Myc-RNF187 or Myc plasmids. After 48 h, cells were harvested for western blot analysis. Myc-RNF187 and YAP protein levels were determined by Western blot. Actin was used as internal control. **d** RNF187 depletion increased YAP target gene expression in TNBC cells. BT549 cells were transfected with siControl or siRNF187. After 48 h, total RNA was extracted for gene expression analysis. Each group was done in triplicates. *P < 0.05; **P < 0.01; ***P < 0.001 for target gene expression comparison. **e** RNF187 overexpression decreased YAP target gene expression in TNBC cells. BT549 cells were transfected with Myc-RNF187 or Myc plasmids. After 48 h, total RNA was extracted for gene expression analysis. Each group was done in triplicates. *P < 0.05; **P < 0.01; ***P < 0.001 for target gene expression comparison.

Following this, we began to analyze the effect of RNF187 in Hippo/YAP signaling. Two independent RNF187 siRNAs shows that RNF187 depletion increases YAP protein level in both BT549 and MDAMB231 cells (Fig. 2a, b). Consistently, transient RNF187 transfection decreases YAP protein level in BT549 cells (Fig. 2c). In further analysis of the Hippo/YAP target genes: CTGF and CYR61. The QPCR data shows that RNF187 depletion increases YAP target gene expression (CTGF and CYR61), while RNF187 overexpression decreases their expression in BT549 cells (Fig. 2d, e).

### RNF187 inhibits cancer cell migration and invasion through Hippo/YAP signaling

**Fig. 3:**
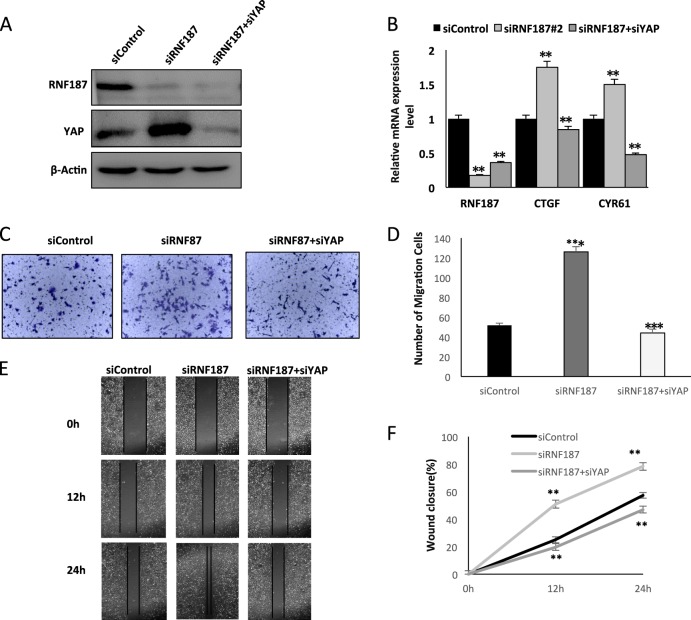
RNF187 inhibits cancer cell migration and invasion through Hippo/YAP signaling. **a** RNF187 depletion increased YAP protein level, which effect could be reversed by YAP knocking-down. BT549 cells were transfected with siControl or siRNF187. After 24 h, cells were transfected with siYAP or siControl. After 48 h, cells were harvested for western blot analysis. RNF187 and YAP protein levels were determined by Western blot. Actin was used as internal control. **b** RNF187 depletion increased Hippo target gene expression, which effect could be reversed by YAP knocking-down. BT549 cells were transfected with siControl or siRNF187. After 24 h, cells were transfected with siYAP or siControl. After 48 h, total RNA was extracted for gene expression analysis. Each group was done in triplicates. *P < 0.05; **P < 0.01; ***P < 0.001 for target gene expression comparison. **c**, **d** RNF187 depletion increased TNBC cell invasion capacity, which effect could be reversed by YAP knocking-down. BT549 cells were transfected with siControl or siRNF187. After 24 h, cells were transfected with siYAP or siControl. After another 24 h, cancer cells were seeded into the chamber for trans-well assay. The cell number was counted and Data are presented as ± SD. **P < 0.01, ***P < 0.001 (student’s *t*-test). **e**, **f** Wound-healing assay indicated that RNF187 depletion increased TNBC cell migration capacity, which effect could be reversed by YAP knocking-down. BT549 cells were transfected with siControl or siRNF187. After 24 h, cells were transfected with siYAP or siControl. Quantification of wound closure at the indicated time points. Data are presented as ±SD. **P < 0.01, ***P < 0.001 (student’s *t*-test).

Having shown that RNF187 can inhibit Hippo/YAP signaling and cell migration and invasion capacity, we experimented further to provide the logic link between cancer cell phenotype and Hippo signaling. Figure 3a shows that increased YAP protein level by RNF187 knocking-down could be rescued by YAP depletion. Besides, RNF187 knocking down could increase Hippo target gene expression, the effect of which could be reversed by YAP depletion in TNBC cells (Fig. 3b). The rescue experiment in trans-well assay was carried out with consistent results: RNF187 depletion significantly promotes cancer cell invasion, which effect is rescued by further YAP knocking down (Fig. 3c, d). In the wound-healing assay, the increased migration capacity of TBNC cells by RNF187 depletion could be rescued by YAP knocking-down (Fig. 3e, f).

### RNF187 reversely correlates with Hippo/YAP signaling in TBNC tumor samples

**Fig. 4:**
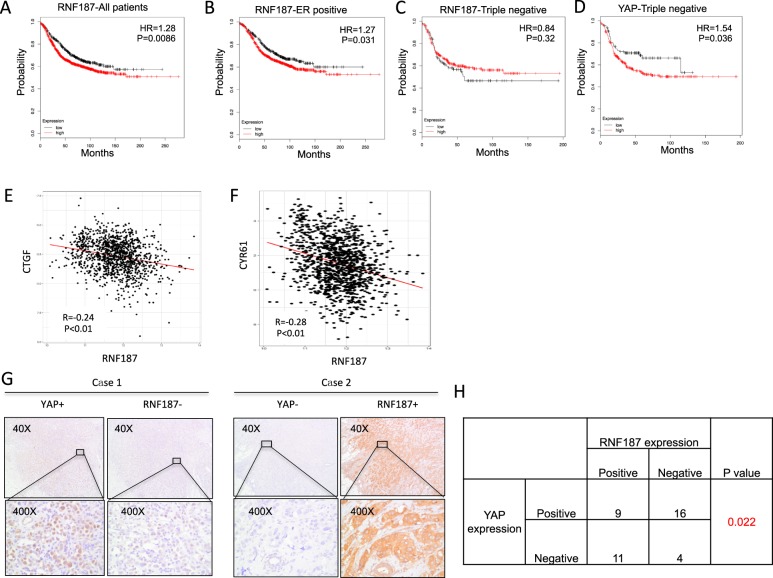
RNF187 reversely correlates with Hippo/YAP signaling in TBNC tumor samples. **a** Kaplan–Meier graph of progression-free survival shows that RNF187 relates to poor prognosis in all breast cancer patients. **b** Kaplan–Meier graph of progression-free survival shows that RNF187 relates to poor prognosis in ER positive breast cancer patients. **c** Kaplan–Meier graph of progression-free survival shows that RNF187 tends to relate to good prognosis in triple negative breast cancer patients. **d** Kaplan–Meier graph of progression-free survival shows that YAP relates to poor prognosis in triple negative breast cancer patients. **e**, **f** Public available data shows that RNF187 is reversely correlated with Hippo/YAP target genes: CTGF and CYR61 (https://www.cbioportal.org). **g** Example tumor cases showing that RNF187 reversely correlates with YAP protein in IHC. **h** Statistical analysis of RNF187 correlation with YAP in forty TNBC tumor samples.

We further analyzed the RNF187 effect in clinical samples. From the KMPLOT (https://kmplot.com), we investigate the prognostic effect of RNF187 in breast cancer patients (Fig. 4a–c). Interestingly, RNF187 has poor prognosis in all breast cancer patients (Fig. 4a). However, RNF187 shows different prognostic trends in different subtype of breast cancer. In ER alpha positive type, RNF187 relates to poor prognosis, while RNF187 shows the trend to be a good prognostic marker (*P* = 0.32) in triple negative breast cancer (Fig. 4b, c). However, YAP expression correlates with poor prognosis in triple negative breast cancer (Fig. 4d). Since we observed that RNF187 shows opposite prognostic trend with YAP in TNBC patients, we further analyzed the expression correlation between RNF187 and Hippo/YAP target genes. From TCGA database, we observe that RNF187 reversely correlates with CTGF/CYR61 expression (Fig. 4e, f). Finally, we collect forty TNBC tumor samples. Immuno-histochemisty shows that RNF187 is localized in the cytoplasm, while YAP is mainly in the nucleus, which is consistent with the public database (https://www.proteinatlas.org). Besides, RNF187 reversely correlates with YAP protein level (Fig. 4g, h).

### RNF187 promotes YAP protein degradation

**Fig. 5:**
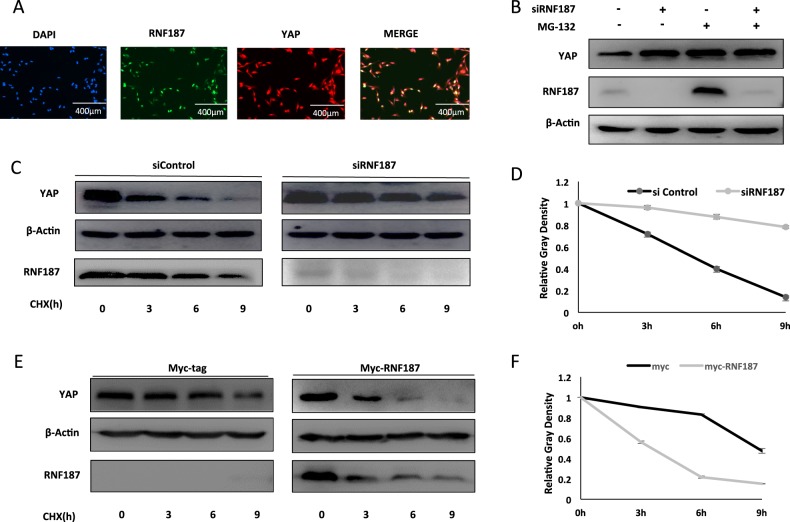
RNF187 promotes YAP protein degradation. **a** Intracellular localization analysis of RNF187 and YAP by immunofluorescence assay. BT549 cells were cultured in normal medium before fixation. Intracellular localization of YAP (red) and RNF187 (green) were shown. Nuclei (blue) were stained with 4′,6-diamidino-2-phenylindole (DAPI). **b** In the presence of the proteasome inhibitor MG132, the degradation effect of RNF187 on YAP did not further increase YAP protein levels. BT549 cells were transfected with siRNF187 or siControl. After 24 h, cells were treated with 10 µM MG132/vehicle for 6 h. Cell lysates were prepared for Western blot analysis. The results are representative for three independent experiments. **c**, **d** RNF187 depletion increased YAP half-life in BT549 cells. BT549 cells were transfected with 50 µM siControl or siRNF187. After 24 h, cells were treated with 100 µM cycloheximide/vehicle for indicated times. Cell lysates were prepared for Western blot analysis. The results are representative for three independent experiments. The YAP relative density was measured by Image J software. **e**, **f** RNF187 decreased YAP half-life in HEK293 cells. HEK293 cells were transfected with 0.5 µg Myc-RNF187 or Myc plasmids. After 24 h, cells were treated with 100 µM cycloheximide/vehicle for indicated times. Cell lysates were prepared for Western blot analysis. The results are representative for three independent experiments. The YAP relative density was measured by Image J software.

We further investigate the localization of RNF187 and YAP in TNBC cell lines. Immuno-staining shows that both RNF187 and YAP are located in the nucleus (Fig. 5a). RNF187 depletion could increase YAP protein level, the effect of which could be reversed by proteasome inhibitor MG132 (Fig. 5b). This could indicate that RNF187 might modulate YAP via protein stability. When we utilize the protein synthesis inhibitor cycloheximide, RNF187 depletion significantly increases the YAP protein half-life in TNBC cells (Fig. 5c, d). Besides, RNF187 overexpression could significantly decrease YAP protein stability in HEK293 cells (Fig. 5e, f).

### RNF187 associates with YAP and promotes YAP K48-linked poly-ubiquitination

**Fig. 6:**
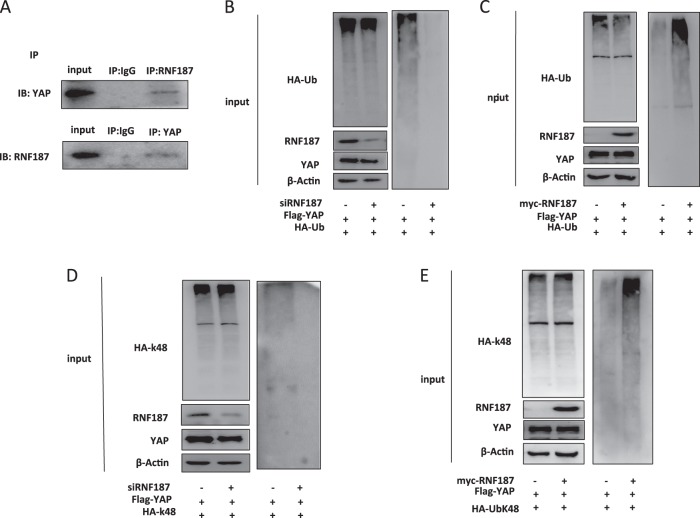
RNF187 associates with YAP and promotes YAP K48-linked poly-ubiquitination. **a** Co-IP assay revealed association between endogenous RNF187 and YAP protein in BT549 cells. BT549 cells were harvested with RIPA lysis buffer. CO-IP was performed using antibody as indicated. **b** RNF187 depletion decreased the overall poly-ubiquitination of YAP. BT549 cells were transfected with 50 µM siControl or siRNF187. After 24 h, cells were transfected with 1 µg HA-Ub plasmid. After another 24 h, the cell extracts were immunoprecipitated with HA antibody. The poly-ubiquitinated YAP was detected via western blotting analysis. **c** RNF187 increased the overall poly-ubiquitination of YAP. HEK293 cells were transfected with 0.5 µg Myc-RNF187 or Myc vector. After 24 h, cells were transfected with 1 µg HA-Ub plasmid. After another 24 h, the cell extracts were immunoprecipitated with HA antibody. The poly-ubiquitinated YAP was detected via western blotting analysis. **d** RNF187 depletion decreased the K48-linked poly-ubiquitination of YAP. BT549 cells were transfected with 50 µM siControl or siRNF187. After 24 h, cells were transfected with 1 µg HA-K48-Ubi plasmid. After another 24 h, the cell extracts were immunoprecipitated with HA antibody. The K48-linked poly-ubiquitinated YAP was detected via western blotting analysis. **e** RNF187 increases K48-linked poly-ubiquitination of YAP. HEK293 cells were transfected with 0.5 µg Myc-RNF187 or Myc vector, together with 1 µg HA-K48 Ubi plasmid. The cell extracts were immunoprecipitated with HA antibody. The K48 specific poly-ubiquitinated YAP was detected via western blotting analysis.

**Fig. 7:**
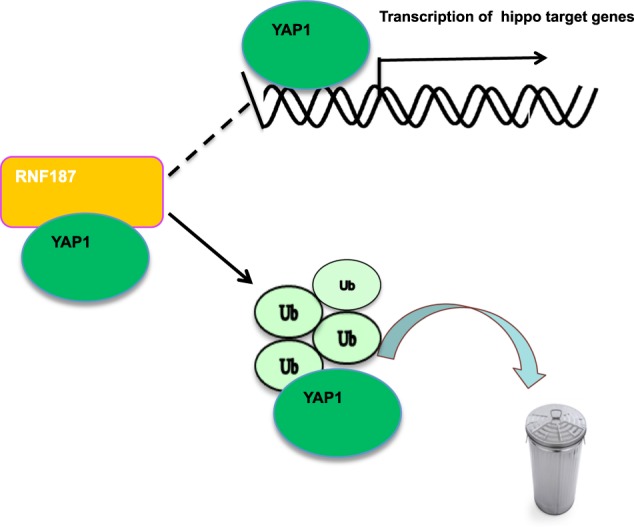
The hypothetical model for RNF187 regulating Hippo signaling in triple negative breast carcinoma. RNF187 protein associated with YAP and promoted YAP degradation via inducing YAP K48-linked poly-ubiquitination.

Functional cooperation between YAP and RNF187 was further supported by additional experimentation. Co-immunoprecipitation shows the endogenous association between RNF187 and YAP in BT549 cells (Fig. 6a). As an ubiquitin ligase, it is most possible that RNF187 modulates YAP protein stability through ubiquitin-dependent manner. We carried out endogenous ubiquitin-based immunoprecipitation assay in BT549 cells, which indicates that RNF187 depletion inhibits YAP poly-ubiquitination (Fig. 6b). However transient over-expression of RNF187 could significantly increase YAP poly-ubiquitination in HEK293 cells (Fig. 6c). Since RNF187 promotes YAP poly-ubiquitination and degradation in TNBC cells, we infer that the ubiquitination modification is proteolytic. Since K48-linked ubiquitin manner is the most common proteolytic ubiquitin modifications, we examine the K48-linked ubiquitination in TBNC cells. The ubiquitin-based immunoprecipitation assay in BT549 cells shows that RNF187 depletion could inhibit YAP K48-linked poly-ubiquitination (Fig. 6d). Consistently, RNF187 overexpression promotes K48-linked poly-ubiquitination in HEK293 cells (Figs. 6e, 7).

## Discussion

In this study, we have identified RNF187 as an E3 ubiquitin ligase, which promotes YAP protein K48-linked ubiquitination and degradation in TNBC cells. Interestingly, RNF187 shows the trend to relate with good prognosis in TNBC (*P* = 0.32). This effect is the opposite in ER+ subgroups. RNF187 shows the opposite prognostic trend with YAP in TNBC; negative correlates with YAP in both protein level and Hippo target gene expression. Our study provides a novel mechanism between RNF187 and Hippo signaling, which could be a promising strategy for TNBC treatment.

Several studies have confirmed the oncogenic role of YAP in modulating tumorigenesis in a group of human cancers^24–28^. For example, YAP gene is amplified in TNBC and liver cancer. The activation of YAP functions to be a transcriptional cofactor and co-activates several transcription factors, such as TEADs, RUNX, and AP1^29,30^, which subsequently promotes cancer cell migration, invasion, and anti-apoptotic effects^31^. When it comes to TNBC, YAP is expressed in higher level in TNBC, while often decreased in ER+ breast cancer^32,33^. Several studies showed that activation of Hippo signaling in TNBC cell lines induces cell proliferation, invasion, anti-apoptotic and stem cell like phenotype. Besides, Hippo/YAP signaling could synergize AP1 family members to promote TNBC cancer progression^30^. In our study, we also showed that YAP expression relates to poor survival in TNBC cells. Based on the current finding and understanding of Hippo function in TNBC cells, targeting YAP could be a plausible way to treat triple negative breast cancer.

YAP protein was firstly identified as a WW domain containing protein, which is composed by three functional domains: TEAD interaction domain, WW domain and transcriptional activation domain^34^. The TEAD interaction domain mediates its interaction with several transcriptional factors and transcriptional activation. However, the on-off status of the YAP function is tightly controlled by several serine and threonine kinases, such as MST1/2 and LATS1/2. The LATS1/2 protein promotes the phosphorylation of YAP at several sites (S61, S109, S127, and S381) and promotes YAP retaining in the cytosol and protein degradation^35^. However, recently studies also put more importance on YAP protein ubiquitination. For example, SCF^b-TRCP^ complex could promote YAP protein poly-ubiquitination and degradation^36^. Besides, FBW7, as an E3 ligase, could also induce YAP protein K48-linked ubiquitination and degradation^37^. In our study, we identified RNF187 as a novel E3 ligase for YAP protein, which suppresses Hippo/YAP signaling by inducing YAP K48-linked ubiquitination and degradation. This finding provides us with a further understanding of the tiny control of YAP protein, as well as offering a new target for blocking hippo signaling in TBNC.

RNF187 protein is composed of two important domains, including RING domain and IRES domain. RING domain is responsible for catalytic activity of E3 ligation, while IRES domain is responsible for interaction with the substrates^20^. One of the interesting finding is that RNF187 involves the regulation of RAS-AP1 signaling^19^. The activation of MEK-ERK pathway promotes the K63-linked ubiquitination of RNF187, which inhibits K48-linked ubiquitination of CDC2 and Cyclin D1 and their degradation^19^. Besides, RNF187 is also the co-factor of AP1 activation and cell proliferation. In our study, this could explain the explanation that in our study why siRNF187 could inhibit cell proliferation in TNBC cells. Although several studies showed that RNF187 could potentially been an oncogene, our understanding is that RNF187 function is cancer type dependent. Our data showed that RNF187 could inhibit TNBC cell invasion via inhibition Hippo signaling. This could be the first study showing the tumor suppressive function of RNF187 in cancer progression. The interesting findings increase the proteolytic regulation of Hippo/YAP signaling, but also reveal the “multi-face” role of RNF187 in different cancer type backgrounds.

Another interesting finding is that both YAP and RNF187 were shown mainly in the nucleus, while the IHC of human samples showed that both of the proteins were localized in the cytosol and nuclear. Although we cannot clarify the reason why the localizations are different in cell lines and human breast cancer samples, this differences are also observed in proteinatlas websites, which shows that RNF187 is mainly localized in the nuclear in cell lines (https://www.proteinatlas.org/ENSG00000168159-RNF187/cell#human), while in the cytosol in human samples (https://www.proteinatlas.org/ENSG00000168159-RNF187/pathology/breast+cancer#img). According to our understanding, the localization of certain protein is relatively consistent in samples, while variable in cell lines. Several factors could affect the localization of protein, such as nutrition. One example is that YAP localizes in the nuclear, while changes into the cytosol under starvation condition^38^. Further investigation is needed to characterize the potential mechanisms, which affects the protein localizations.

In conclusion, this study identifies for the first time, RNF187 as a modulator of Hippo signaling in human TNBC cells. RNF187 depletion promotes cancer cell progression and activates Hippo signaling in multiple TNBC cell lines. As a modulator for Hippo signaling, modulation of RNF187 activity or expression level could be a promising approach to treat TNBC.

## Materials and methods

### Cell culture

BT549, MDAMB231, and HEK293 cells were ordered form American Type Culture Collection (ATCC). HEK293 cells were cultured in Dulbecco’s Modified Eagle’s Medium that contains 4.5 g/L glucose and 4 mM l-glutamine (DMEM, 41965, Life Technologies) supplemented with 10% Fetal Bovine Serum (FBS, 10270, Life Technologies). MDAMB231 and BT549 cells grown in RPMI-1640 (42401, Life Technologies) supplemented with 2 mM l-glutamine (25030, Life Technologies) and 10% FBS. All cell lines were subject to cell line authentication. The cell line authentication via Short Tandem Repeat (STR) was performed via PowerPlex 21 system. The STR data of HEK293, MDAMB231, and BT549 cell lines were found consistent with STR data in ATCC.

### Plasmids and siRNA

The Flag-RNF187 plasmid was acquired from Origene. The The HA-K48 and HA-K63 Ubi plasmids were acquired from our previous study^39^. The Lipofectamin 2000 (1662298, Invitrogen) was used for the plasmids transfection. Small interfering RNAs were used for specific gene knocking-down. The RNF187 siRNA sequences were: GUGAUGGACCGUAGGAAGAdTdT; UCUUCCUACGGUCCAUCACdTdT and CACUGAGCGGUUCAGGUCAdTdT; UGACCUGAACCGCUCAGUGdTdT. The YAP siRNA sequence were: GCUCAUUCCUCUCCAGCUUTT; AAGCUGGAGAGGAAUGAGCTT. The negative control siRNA sequences were: UUCUCCGAACGUGUCACGUTT; ACGUGACACGUUCGGAGAATT. The RNAiMAX reagent (13778150, Invitrogen) was used for siRNA transfection.

### RNA extraction and qPCR analysis

RNeasy plus mini kits were used to extract total RNA (Qiagen). The RNA concentration was measured via Nanodrop. The RNA quality was pre-checked via 18S/28S ratio in 1% agarose gel. Real-time PCR was performed as previously described. 36B4, beta-actin, and GADPH were used for internal control^40^. The relative gene expression levels were calculated and normalized with the geometric mean of the three controls. The primer sequences are shown here. RNF187: F: agg act tga atg acg ccc g; R: tcc atc acg tgt ccc ttc ca. 36B4: F: ggc gac ctg gaa gtc caa ct; R: cca tca gca cca cag cct tc. CTGF: F: ctc gcg gct tac cga ctg; R: ggc tct gct tct cta gcc tg. CYR61: F: agc agc ctg aaa aag ggc aa; R: agc ctg tag aag gga aac gc. Beta-actin: F: aca gag cct cgc ctt tgcc; R: gat atc atc atc cat ggt gag ctgg. GAPDH: F: tcg gag tca acg gat ttg gt; R: ttc ccg ttc tca gcc ttg ac.

### Quantification of cell viability

MDAMB231 and BT549 cells were transfected with siRNF187 or siControl in 24-well plate. Twenty-four hours after transfection, the cells number was countered and 4000 cells were seeded into 96-well plates. The relative cell viability was measured at indicated time points. Cell numbers were determined using the WST-1 cell proliferation reagent as previously described^41^.

### Wound healing assay

MDAMB231 and BT549 cells were transfected with 50 µM RNF187 siRNA or sControl. After 24 h, cells were seeded into 12-well plates with 1% FBS. The cells were 100% confluence. The yellow pipette tips were applied for straight scratch. The wound distance was measured at indicated time points and normalized with starting time point. The wound healing recovery was expressed as: [1 − (Width of the wound at a given time/width of the wound at *t* = 0)] × 100%.

### Trans-well assay

Cell invasion capacity was measured using the modified two-chamber plates as before. For invasion assay MDAMB231 cells and BT549 cells were were transfected with 50 µM RNF187 siRNA or sControl. In order to stimulate invasion, the bottom wells were filled with complete medium, while the upper chambers were added with FBS-free medium. After 12 h, cells were carefully removed and the cells that invaded through the membrane were fixed and stained with Crystal Violet Staining solution. The cell numbers were counted by using a microscope.

### Clone formation assay

MDAMB231 and BT549 cells were seeded in six-well plates overnight and treated with 50 nM RNF187 siRNA or 50 nM siControl. Twenty-four hours post-transfection, the cells were washed with PBS, trypsinized and plated at low density (5000 cell/well in six-well plate). The cells were cultured for 10 days and the medium was refreshed every 2 days. The colonies were stained with crystal violet. The number of the clones in a given area was counted for each condition.

### Western blotting

Cells were harvested and lysed with RIPA buffer. Proteins were separated by electrophoresis on SDS-polyacrylamide gel electrophoresis (PAGE) and electro-transferred to PVDF membrane. The antibodies used in this study were listed here: Anti-RNF187 (HAP030098, Sigma); Anti-YAP (SC-101199, Santa Cruz); Anti-HA (MMS-101R, COVANCE); Anti-myc (9E10, ab32, Abcam); Anti-myc (Ab9106, Abcam); Anti-GAPDH (GB12002, Servicebio); Anti-Flag (20543-1-AP, Proteintech); Anti-GFP (Ab290, Abcam). For western blot assays, the antibodies were used in the following concentration: Anti-YAP (SC-101199, Santa Cruz): 1/2000; Anti-HA (MMS-101R, COVANCE): 1/1000; Anti-myc (9E10, ab32, Abcam): 1/1000; Anti-RNF187 (HAP030098, Sigma): 1/1000; Anti-Flag (20543-1-AP, Proteintech): 1/1000; Anti-GFP (Ab290, Abcam): 1/1000. Membranes were then washed with PBS for three times and incubated with secondary antibodies Peroxidase-Conjugated AffiniPure Goat Anti-Mouse IgG or Goat Anti-Rabbit IgG. Fluorescent signals were visualized with ECL system. (amersham imager 600, USA).

### Co-immunoprecipitation assay

Immunoprecipitation was performed as described in the previous study. The BT549 cells total cell lysls were pre-cleared with rabbit IgG for 2 h and subsequently immunoprecipitated with RNF187 antibody (HAP030098, Sigma, 1/200) or YAP antibody (SC101199, santa cruz, 1/200) over night, while rabbit IgG (Santa Cruz) was used as the negative control. The bounded protein was analyzed by Anti-YAP antibody (SC-101199, Santa Cruz; 1/1000) or RNF187 antibody (HAP030098, Sigma, 1/1000).

### Protein stability assays

About 10^5^ HEK293 cells were seeded into twenty-four well plates and transfected with 0.5 µg Flag-RNF187 or Flag-vector. After 48 h, cells were treated with 100 µM cycloheximide (C7698, Sigma) for indicated time points. Samples were subject to western blot for YAP degradation. For MDAMB231 cells, 105 cells were seeded into 24 well-plate and transfected with 50 nM siRNF187 or siControl. After 24 h, cells were treated with 100 µM cycloheximide (C7698, Sigma) for indicated time points. Samples were subject to western blot for YAP degradation.

### Poly-ubiquitination detection assay

To directly detect the enriched K48-ubiquitinated and K63-ubiqutinated YAP from the cell extracts, HEK293 cells were transfected with 0.5 µg K48 Ubi or 4 µg K63 Ubi plasmids together with 0.5 µg Flag-RNF187 or Flag-vector. After 48 h, the total protein was extracted and pre-cleared with 20 µL protein A (santa cruz, SC-2001) for 2 h. The supernatant was collected and immunoprecipitated by YAP antibody. Western blot with HA antibody was performed to detect K48 or K63 poly-ubiquitinated YAP.

### Immunofluorescence assay

EC109 cells were fixed with 4% paraformaldehyde in PBS for 10 min, permeabilized with 0.2% Triton X-100 for 5 min, and blocked by 5% BSA in PBS for 1 h. A rabbit anti-RNF187 polyclonal antibody (HAP030098, Sigma, 1/50) and mouse anti-YAP monoclonal antibodies (SC-101199, Santa Cruz, 1/50) were used, followed by Alexa Flour 647 (Invitrogen) anti-rabbit antibody and FITC-conjugated anti-mouse antibodies (Jackson ImmunoResearch, West Grove, PA). As negative controls, the samples were incubated with the secondary antibodies without primary antibodies. Images were acquired under conditions fulfilling the Nyquist criterion using Nikon A+ laser scanning confocal system with a 60× oil NA1.4 objective and pinhole size of 1.0 Airy Unit. The acquired pictures were further processed and assembled using ImageJ.

### Public available clinical data analysis

Analysis of RNF187 and YAP correlation with clinical prognosis was carried out through KMPLOT database (https://kmplot.com). Analysis of RNF187 correlation with YAP target gene (CTGF and CYR61) was carried out by TCGA database with 1080 breast cancer samples. The analysis was carried out with “ggcorrplot” package in the statistical environment R-3.6.1 version. The “ggcorrplot” package version is “ggcorrplot 0.1.3”. The detailed information of “ggcorrplot” can be found in the following link: https://github.com/kassambara/ggcorrplot.

### Clinical breast tumor samples

Forty triple negative breast cancer samples were collected from QIlu Hospital of Shandong University. All the triple negative breast cancer samples were examined in ER, PR, and HER2 status. The immuno-histochemisty of RNF187 and YAP were carried out according to standard method. The IHC results of RNF187 and YAP were examined through pathological specialists. The rabbit anti-RNF187 polyclonal antibody (HAP030098, Sigma, 1:100) and mouse anti-YAP monoclonal antibodies (SC-101199, Santa Cruz, 1/100) were used for IHC analysis. The size of the FFPE section was prepared in 4 μm. The results of YAP/RNF187 staining were determined by two independent certified pathologists.

### Statistics

Student’s *t*-test, Pearson correlation coefficient, and Cox regression analysis were used for comparisons. A *P*-value of <0.05 was considered to be significant.

## Supplementary information

Cell line authentication

Cell line authentication

## Data Availability

The public available data is in the supplementary materials.
