# Supplementary material for: Regulation of Hippo signaling and triple negative breast cancer progression by an ubiquitin ligase RNF187
Source: Oncogenesis. 2020 Mar 20;9(3):36. doi: 10.1038/s41389-020-0220-5 (PMC7083878; doi:10.1038/s41389-020-0220-5)
Supplement: Supplementary file 1 — Cell line authentication [file 41389_2020_220_MOESM1_ESM.pdf]

MDA-MB-231 细胞 STR 鉴定报告

一、 材料处理和检验方法

取适量 **MDA-MB-231** 细胞(1×10^{6})使用 PureLink® Genomic DNA Mini Kit（美国 Life K182001）提前基因组 DNA，采用 PowerPlex®18D 系统（美国 Promega DC1802）试剂盒进行扩增，在 ABI3500 Genetic Analyzer（美国 Life3500）进行检测。

二、 检测结果

实验中阴性及阳性对照结果均正确。

MDA-MB-231 细胞株的 STR 位点和 Amelogenin 位点的基因分型结果见附表，分型图谱见附图。

三、 分析说明

MDA-MB-231 细胞株基因组 DNA 扩增后图谱清晰，分型结果良好。

四、 检验结论

1. **MDA-MB-231** 细胞株 DNA 进行细胞 STR 分型结果显示，细胞株中未发现人类细胞交叉污染。
2. 该细胞株 DNA 分型在 ATCC 细胞库中找到与其细胞分型 100%相匹配的细胞株，细胞株名称为 **MDA-MB-231**。

Procell
www.procell.com.cn

附表 1：细胞株 **MDA-MB-231** 的 **STR** 位点和 **Amelogenin** 位点的基因分型结果

细胞 **MDA-MB-231**（图片编号为 PC03）		
Marker	***Allele 1***	***Allele 2***
D3S1358	16	16
**THO1**	7	9.3
D21S11	33.2	33.2
D18S51	11	16
Penta E	11	11
**D5S818**	12	12
**D13S317**	13	13
**D7S820**	8	9
**D16S539**	12	12
**CSF1PO**	12	13
Penta D	11	14
**AMEL**	X	X
**Vwa**	15	18
D8S1179	13	13
**TPOX**	8	9
FGA	22	23

附图 1：**ATCC** 官网 **MDA-MB-231** 细胞 **STR** 位点信息

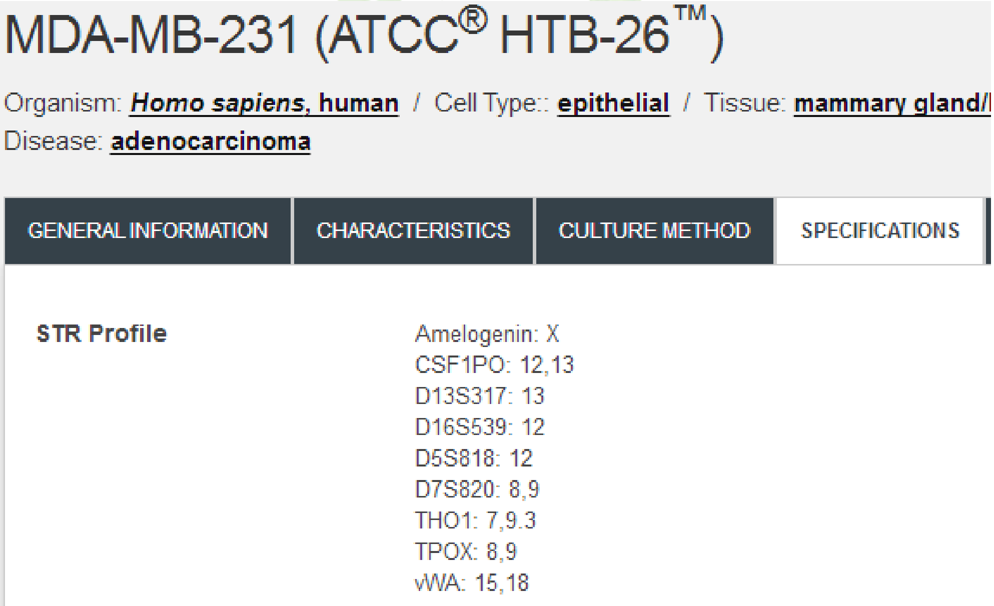

附图 2：**MDA-MB-231** 细胞 **STR** 位点和 **Amelogenin** 位点的基因分型结果

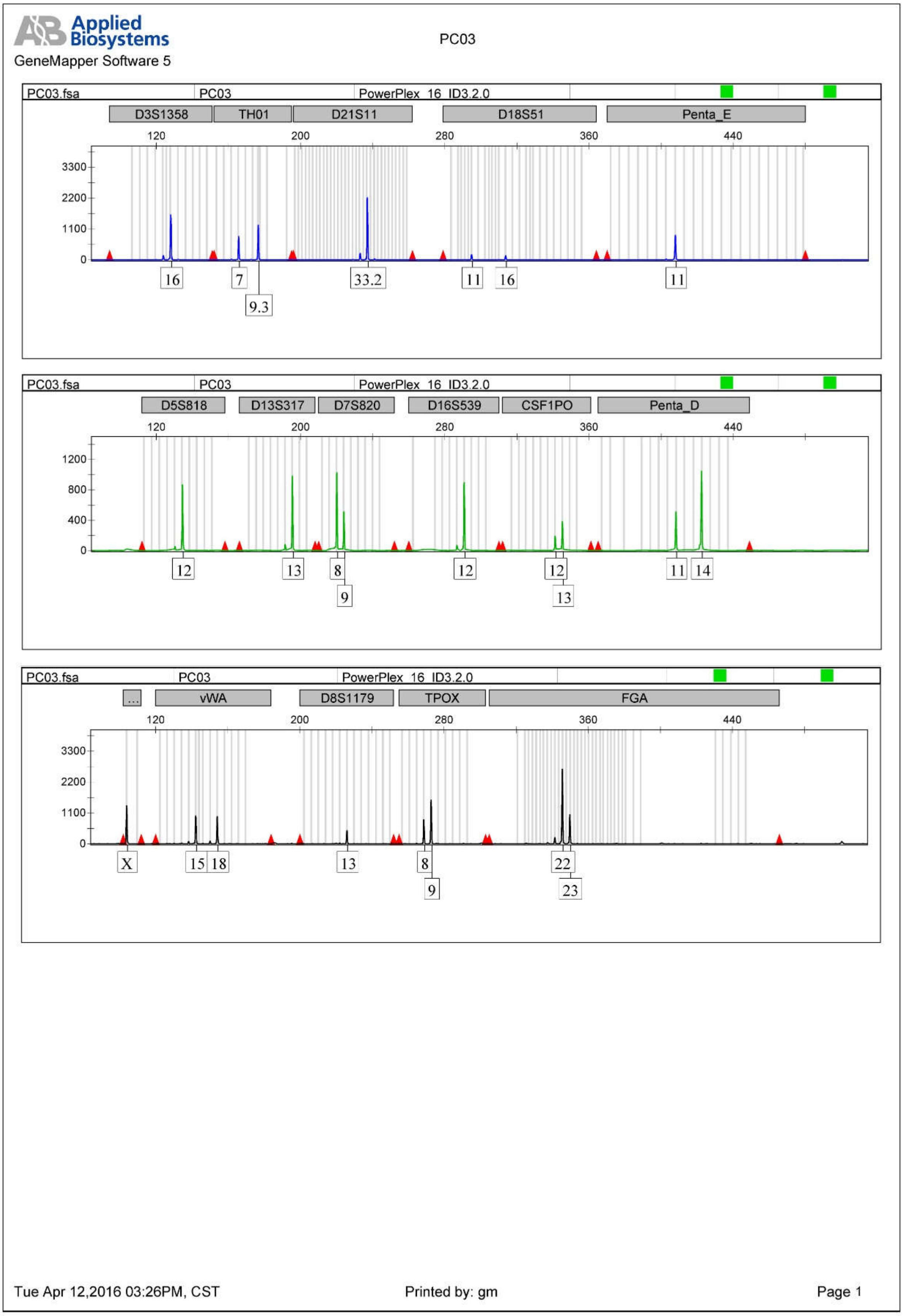

网站：www.procell.com.cn
电话：400-650-3656
邮箱：sales@procell.com.cn
