# Supplementary material for: Regulation of Hippo signaling and triple negative breast cancer progression by an ubiquitin ligase RNF187
Source: Oncogenesis. 2020 Mar 20;9(3):36. doi: 10.1038/s41389-020-0220-5 (PMC7083878; doi:10.1038/s41389-020-0220-5)

BT-549 细胞 STR 鉴定报告

一、 材料处理和检验方法

取适量 **BT-549** 细胞(1×10^6)使用 PureLink® Genomic DNA Mini Kit（美国 Life K182001）提前基因组 DNA，采用 PowerPlex®18D 系统（美国 Promega DC1802）试剂盒进行扩增，在 ABI 3500 Genetic Analyzer（美国 Life 3500）进行检测。

二、 检测结果

实验中阴性及阳性对照结果均正确。

BT-549 细胞株的 STR 位点和 Amelogenin 位点的基因分型结果见附表，分型图谱见附图。

三、 分析说明

BT-549 细胞株基因组 DNA 扩增后图谱清晰，分型结果良好。

四、 检验结论

1. **BT-549** 细胞株 DNA 进行细胞 STR 分型结果显示，细胞株中未发现人类细胞交叉污染。
2. 该细胞株 DNA 分型在 ATCC 细胞库中找到与其细胞分型 100%相匹配的细胞株，细胞株名称为 **BT-549**。

www.procell.com.cn

附表 1：细胞株 BT-549 的 STR 位点和 Amelogenin 位点的基因分型结果

细胞 **BT-549** 图片编号为 PC165）				
STR Loci	***Allele 1***	***Allele 2***	***Allele 3***	***Allele 4***
**Amelogenin**	X	X		
**D3S1358**	18	18		
**D1S1656**	12	17.3		
**D6S1043**	11	11		
**D13S317**	11	11		
**Penta E**	14	14		
**D16S539**	8	8		
**D18S51**	15	15		
**D2S1338**	17	17		
**CSF1PO**	10	12		
**Penta D**	13	13		
**TH01**	9.3	9.3		
**vWA**	15	18		
**D21S11**	32.2	32.2		
**D7S820**	9	10		
**D5S818**	11	11		
**TPOX**	8	8		
**D8S1179**	16	16		
**D12S391**	18	20		
**D19S433**	15.2	15.2		
**FGA**	19	19		

附图 1：ATCC 官网 BT-549 细胞 STR 位点信息

BT-549 (ATCC® HTB-122™)

Organism: ***Homo sapiens*, human** / Cell Type: **Epithelial** / Tissue: **mammary gland;**

GENERAL INFORMATION | CHARACTERISTICS | CULTURE METHOD | SPECIFICATIONS

STR Profile

Amelogenin : X
CSF1PO: 10 , 12
D13S317: 11
D16S539 : 8
D5S818: 11
D7S820: 9, 10
TPOX: 8
THO1: 9.3
vWA : 15

网站：www.procell.com.cn
电话：400-650-3656
邮箱：sales@procell.com.cn

www.procell.com.cn

附图 2：BT-549 细胞 STR 位点和 Amelogenin 位点的基因分型结果

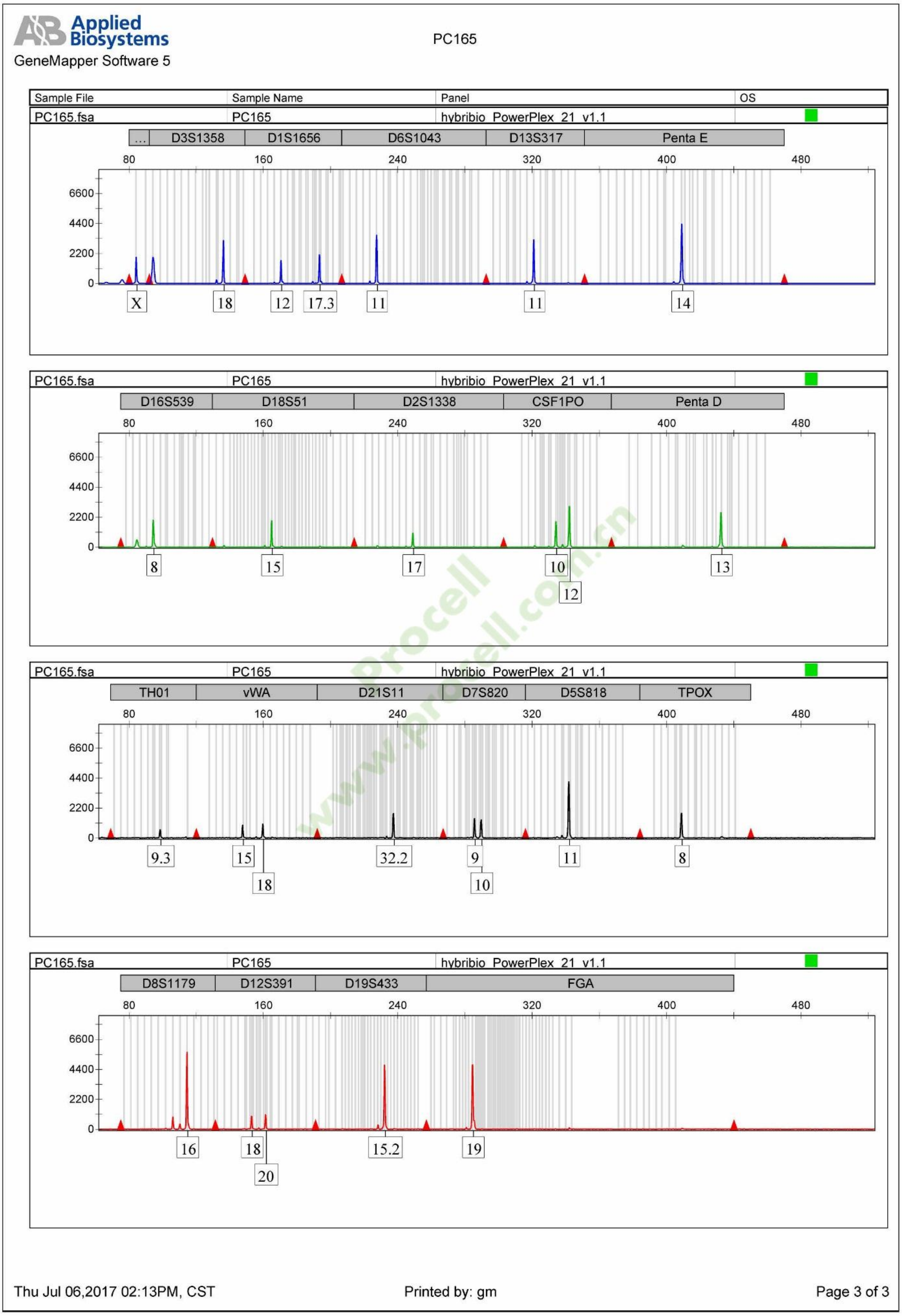

Supplement: Supplementary file 2 — Cell line authentication [file 41389_2020_220_MOESM2_ESM.pdf]
